# Global Health and Public Health Majors and Minors at 411 Universities, 2019–2020

**DOI:** 10.5334/aogh.2837

**Published:** 2020-06-19

**Authors:** Caryl E. Waggett, Kathryn H. Jacobsen

**Affiliations:** 1 Department of Global Health Studies, Allegheny College, Meadville, PA, US; 2 Department of Global and Community Health, George Mason University, Fairfax, VA, US

## Abstract

**Background:**

There has been rapid growth in the popularity of undergraduate degrees in global and public health, but that growth has not been evenly distributed across different types of institutions of higher education.

**Objective:**

To examine the prevalence of undergraduate global and public health majors and minors and related degrees at a diversity of higher education institutions in the United States during the 2019–20 academic year.

**Methods:**

We examined curricular offerings at the top 100 national universities, national liberal arts colleges, regional universities, and regional colleges included in the 2020 *U.S. News and World Report* rankings. With ties, the dataset included 411 of the 1600 ranked U.S. colleges and universities.

**Findings:**

In total, 101 (25%) of the 411 schools offer a general public health, community health, or global health major, 105 (26%) a minor, and 144 (35%) a major and/or minor. When subdisciplines and other population health related programs are included, 160 (39%) offer a major, 183 (45%) a minor, and 227 (55%) a major and/or minor, including 83% of national universities, 57% of regional universities, 45% of national liberal arts colleges, and 35% of regional colleges. Global health programs, usually minors, are offered by 32% of national universities and 8% of national liberal arts colleges.

**Conclusions:**

Global and public health have become common areas of primary and secondary study at the bachelor’s level at diverse schools in the United States. Although these degree pathways are especially prevalent at large urban universities, schools of all sizes, types, and locations have invested in offering educational programs in population health areas.

## Introduction

Undergraduate public health education has been growing in popularity since the start of the 21^st^ century when national initiatives such as the Educated Citizen and Public Health program of the Association of American Colleges & Universities began promoting public health as an area of study that engages students with the liberal arts and sciences, develops practical and critical thinking skills, and encourages personal and social responsibility [1]. Today, public health majors and minors typically require coursework in areas identified by the Council on Education for Public Health as critical domains for baccalaureate public health programs, including biological and environmental sciences, social and behavioral sciences, health policy and governance, statistics, and health communication [2]. Some colleges and universities offer global health majors and minors that emphasize globalization and international health disparities rather than focusing primarily on domestic health issues in the United States [3]. Other schools offer curricula in other areas related to population health.

The number of schools offering undergraduate public health majors doubled between 1992 and 2007, doubled again between 2007 and 2012, and has continued to increase [4]. However, the growth in popularity of education in population health areas at the baccalaureate level has not been evenly distributed across different types of institutions of higher education. Most research on the growth in enrollment in public and global health courses and programs has focused on schools with highly competitive application processes rather than institutions that admit the majority of their applicants [5,6,7]. Colleges and universities with different institutional missions and resources may have different levels or types of engagement with public health, global health, and other dimensions of population health. In this article, we examine the prevalence of majors and minors in public health, global health, and related fields at a diversity of higher education institutions, including national and regional universities as well as national liberal arts colleges and regional colleges.

## Methods

### Inclusion criteria

The *Best Colleges 2020* report published by *U.S. News and World Report (USNWR)* in September 2019 categorized 1600 bachelor’s degree granting institutions into four categories derived from the Carnegie Classification system, and then ranked the institutions within each of those groupings based on measures of reputation and quality. Two of the four categories are for universities that offer graduate degrees and two are for colleges that emphasize undergraduate education. National universities are Carnegie “doctoral universities” that offer undergraduate, master’s, and doctoral degrees. Regional universities are Carnegie “master’s colleges and universities” that offer undergraduate degrees, some master’s degree programs, and few or no doctoral programs. Colleges are divided by whether most majors are awarded in arts and sciences disciplines or in professional areas such as business, engineering, nursing, social work, and teaching. National liberal arts colleges are Carnegie “baccalaureate colleges” with an “arts and science focus” that award at least half of their degrees in traditional liberal arts disciplines. Regional colleges are “baccalaureate colleges” or “baccalaureate/associate’s colleges” that award degrees in “diverse fields,” with fewer than half of their degrees awarded in traditional liberal arts fields.

To generate a sample of colleges and universities from various types of four-year institutions of higher education in the United States, we identified the top 100 national universities and the top 100 national liberal arts colleges for inclusion in our analysis. Regional universities and colleges were ranked within four geographic zones defined by state boundaries: North, South, Midwest, and West. We selected the top 25 regional universities and top 25 regional colleges from each of the four geographic zones. When schools receive identical scores, *USNWR* includes tied institutions in their “top 100” and “top 25” lists. Because of those extra lines in the ranking tables, the total number of included schools was 411 rather than 400.

### Data collection

We acquired location, enrollment, and other information about each of the 411 institutions from the Carnegie Classifications Data 2018 public data file (version 7, released 24 May 2019) produced by the Indiana University Center for Postsecondary Research. In this data file, schools are classified as “more selective” if the 25^th^ percentile of SAT and/or ACT scores among first-year students is among the top 20% of all baccalaureate institutions’ entrance examination scores. “More selective” schools typically have an acceptance rate of 55% or less. “Selective” schools generally have standardized scores in the 40^th^ to 80^th^ percentile and admissions rates of about 55% to 80%. “Inclusive schools” generally accept at least 80% of applicants or have open admissions. The degree of urbanization for a school—that is, whether it is in a city, a suburb, or a rural location—is derived from U.S. Census Bureau classifications.

In August and September 2019, we searched each school’s official institutional website to identify educational programs that met our eligibility criteria and appeared to be offered during the 2019–20 academic year. We did not rely on CIP codes, but instead used a systematic search process to gather information about each program. We used several different approaches to ensure the completeness of this search. We began by searching each institution’s website for a list of undergraduate majors and minors. We then used additional search methods to validate our classifications, including searching institutional websites and catalogs for terms such as “public health” and “global health,” using a general search engine to search for institutional names name and key terms, and searching databases of educational programs for schools offering health-related majors. These databases included the College Navigator program from the National Center for Education Statistics, the profiles available on the *USNWR* website, and membership lists from Council on Education for Public Health (CEPH) and the Consortium of Universities for Global Health (CUGH).

### Eligible program areas

We searched for educational programs in public health (PH) and global health (GH) as well as those that were public health related (PHR) and global health related (GHR). Programs in “public health” and “community health” were classified as PH programs. Programs in other areas closely related to public and community health, such as “health promotion” and “health and society” were classified as PHR programs. Programs in “global health” and “global public health” were classified as GH programs. Programs in other areas closely related to global and international health were classified as GHR programs. A full list of categorizations is provided in Table 1. We also considered bachelor’s programs that were CEPH-accredited or candidates for CEPH accreditation to be public health majors even if the name (such as “health science”) was not included in the list of eligible program names in Table 1.

**Table 1:** Classifications for major and minor programs in public health, global health, and related fields.


Public Health	Community and Behavioral Health Community Health Community Health Education Community Health Promotion Health Promotion and Disease Prevention Studies Health Promotion and Science Population and Public Health	Population Health Public and Community Health Public Health Public Health and Education Public Health and Health Equity Public Health Education Public Health Science(s) Public Health Studies
Global Health	Community and Global Health Community and Global Public Health Global and Comparative Public Health Global and Public Health Science Global Health Global Health and Health Policy	Global Health Promotion Global Health Studies Global Health, Culture, and Society Global Public Health Global Public Health and Epidemiology Public Health Global Health
Public Health Related	*To be considered “public health related,” a major or minor had to be in a traditional discipline within public health (such as environmental health or epidemiology), focus on health and society or related areas (such as medical sociology or medical humanities), or otherwise include required courses on community or public health. Programs in areas such as behavioral health, health education, health promotion, health sciences, and health studies were excluded if they required only premedical science courses, were teacher credentialing programs, or were in other excluded program categories*.	
Global Health Related	*Majors and minors in subareas within global health were considered to be “global health related.” This included programs in biology of global health, global disease biology, global health affairs, global health and humanitarian assistance, global health technologies, international health and development, language and international health, medical anthropology, and medical geography in global health*.	


For simplicity, the term “population health” will be used in this paper to refer to overall analysis of public health and global health programs together, since both areas of study focus on determinants of health, health outcomes, and health policies and interventions for groups of people rather than individuals [8]. Population health was not a commonly used name for undergraduate majors or minors during the 2019–20 academic year, but this term appears to be growing in popularity. Public health majors often have an applied practice focus akin to the professional preparation offered at the undergraduate level in areas such as education, nursing, and social work. By contrast, population health does not currently align with one specific career pathway or training area.

### Ineligible program areas

Our goal was to keep the focus on population health programs, so we excluded the following study areas from consideration: (1) applied health science programs focused on individual health rather than public health, such as athletic training, coaching, exercise science, fitness, health and human performance, human development, kinesiology, nutrition, and sports studies; (2) business programs, such as healthcare administration, healthcare management, and health information systems; (c) clinical preparation programs, such as counseling, dietetics, nursing, and speech pathology; (d) educational licensure tracks, such as physical education and school health; (e) general science, technology, and engineering programs, such as biology, biomedical engineering, clinical and medical laboratory sciences, and environmental science; (f) general social science and social service programs, such as anthropology, community development, global studies, human services, international development, international relations, public policy, and psychology, and (g) pre-clinical curricular tracks, such as pre-medical, pre-physical therapy, and pre-occupational therapy studies. Majors and minors with the word “health” in their names, such as “environmental health” and “health and society” were considered for inclusion, but general majors and minors that did not include health in the title, such as “environmental studies” and “science, technology, and society,” were not eligible for inclusion. When it was not clear whether a program with health, medicine, or related terms in the program name had a population focus, we examined the curriculum to determine whether it aligned with one of the ineligible areas above or had a general population health focus.

### Eligible programs types

We divided bachelor’s-level majors, minors, concentrations, fields, certificates, clusters, and other curricular pathways into two categories for analysis. Majors were defined as primary areas of study in a bachelor’s degree program, typically requiring completion of a curriculum that includes 40 to 65 credit hours or an equivalent number of courses in the study area. Minors were defined as secondary programs, typically requiring completion of approximately 15 to 26 credits hours or the equivalent. These types of primary and secondary programs were classified as majors and minors even for schools that used other terms to describe their curricular pathways. We excluded from analysis concentrations within non-health majors (such as anthropology majors with concentrations in global health) and curricular pathways requiring fewer credits than a minor.

## Results

### Number of schools offering population health programs

**Figure 1:**
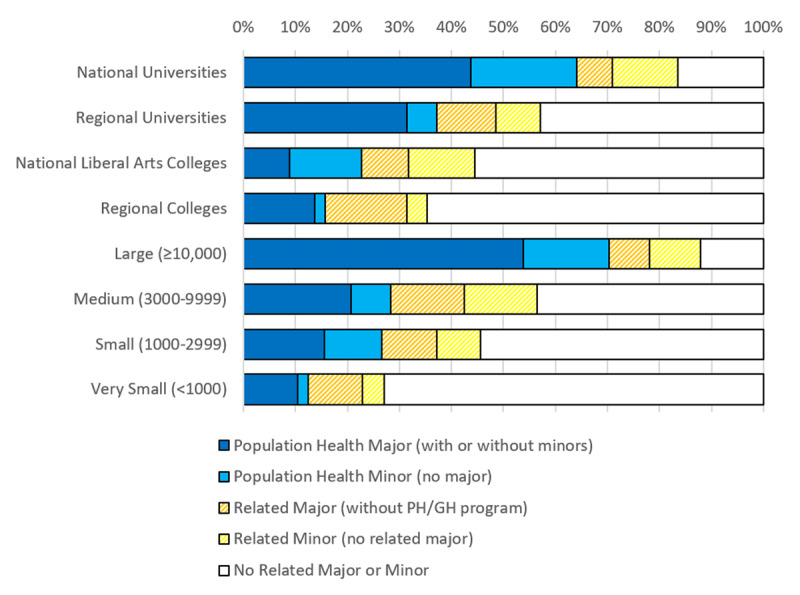
Distribution of schools offering majors and minors in public health and/or global health (blue) and in related fields (yellow), by institutional type and enrollment during the 2019–20 academic year.

In total, 101 (25%) of the 411 schools offer a major in a population health area, 105 (26%) offer a minor, and 144 (35%) offer a major and/or minor (Table 2A). When majors and minors in related areas are added to the analysis, 160 (39%) of the 411 schools offer a major, 183 (45%) offer a minor, and 227 (55%) offer a major and/or minor (Figure 1). Educational programs in population health areas are most prevalent at doctoral degree granting universities and least prevalent at undergraduate-focused colleges that award most of their degrees in professional areas (such as business) rather than the liberal arts and sciences.

**Table 2A:** Number of institutions in the United States that are among the “top 100” national universities, regional universities, liberal arts colleges, or regional colleges evaluated by *US News and World Report* during the 2019–20 academic year and offer majors and minors in public health, global health, and related fields.

		National Universities		Regional Universities		National Liberal Arts Colleges		Regional Colleges		Total

		#	%	#	%	#	%	#	%	#

Total in *USNWR* rakings		399	–	605	–	223	–	373	–	1600
Number in “top 100,” including ties		103	–	105	–	101	–	102	–	411
Public Health (PH)	Major	39	38	32	30	7	7	14	14	92
	Minor	28	27	21	20	13	13	12	12	74
	Major and/or Minor	46	45	38	36	15	15	15	15	114
Global Health (GH)	Major	10	10	1	1	2	2	0	0	13
	Minor	30	29	2	2	8	8	0	0	40
	Major and/or Minor	33	32	3	3	8	8	0	0	44
Population Health (PH and/or GH)	Major	45	44	33	31	9	9	14	14	101
	Minor	50	49	22	21	21	21	12	12	105
	Major and/or Minor	66	64	39	37	23	23	16	16	144
Population Health and/or related field	Major	62	60	46	44	20	20	32	31	160
	Minor	72	70	46	44	39	39	26	25	183
	Major and/or Minor	86	83	60	57	45	45	36	35	227

### Characteristics of schools with population health programs

Total enrollment is strongly correlated with population health offerings (Table 2B). Among the 91 large institutions in the sample, 54% offer a major, 51% a minor, and 70% a major and/or minor. Only 28% of the 92 medium schools and 27% of the 180 small schools offer a major and/or minor. Only 13% of 48 very small schools do. Large comprehensive universities with sizable graduate student populations are the most likely to offer population health majors and minors. Of the 77 doctoral universities classified as having very high research levels, 49% offer a major, 52% a minor, and 71% a major and/or minor. Among the 54 comprehensive doctoral universities in the sample that grant a medical degree (MD, DDS, DMD, DO, DVM and/or), 63% offer a major, 59% a minor, and 83% a major and/or minor. Among the 57 total schools in the sample with CEPH-accredited Master of Public Health (MPH) programs, 63% offer a major, 63% a minor, and 88% a major and/or minor. Exclusively undergraduate institutions are the least likely to offer population health degrees, with only 10% offering a major, 18% a minor, and 20% a major and/or minor.

**Table 2B:** Institutional characteristics of colleges and universities offering majors and minors in public health and/or global health in the United States during the 2019–20 academic year. Rows within each category are ordered based on the percentage shown in the final column.

		Total	Major		Minor		Major and/or Minor	

		#	#	%	#	%	#	%

Total		411	101	25	105	26	144	35
Size classification by full-time equivalent enrollment (2018)	Large (≥10,000)	91	49	54	46	51	64	70
	Medium (3000–9999)	92	19	21	16	17	26	28
	Small (1000–2999)	180	28	16	39	22	48	27
	Very small (<1000)	48	5	10	4	8	6	13
Undergraduate students as a percentage of total enrollment (2018)	Majority graduate (<50%)	17	5	29	8	47	10	59
	Majority undergraduate (50% to 74%)	63	26	41	27	43	35	56
	High undergraduate (75% to 89%)	98	32	33	32	33	43	44
	Very high undergraduate (≥90%)	137	28	20	21	15	37	27
	Exclusively undergraduate (100%)	96	10	10	17	18	19	20
Undergraduate selectivity (2018)	More selective (80^th^ to 100^th^ percentile)	246	66	27	74	30	100	41
	Selective (40^th^ to 80^th^ percentile)	118	24	20	22	19	32	27
	Inclusive	43	10	23	8	19	11	26
Ownership	Public	96	39	41	34	35	49	51
	Private	315	62	20	71	23	95	30
Location	City: midsize	49	21	43	19	39	27	55
	City: large	87	24	28	30	34	37	43
	City: small	52	16	31	14	27	21	40
	Town	96	20	21	25	26	30	31
	Suburb	106	19	18	16	15	27	25
	Rural	21	1	5	1	5	2	10

Schools with more selective undergraduate admissions criteria are more likely to offer population health programming than less competitive schools. While 41% of the 246 “more selective” schools in our sample offer a major and/or minor, only 27% of the 118 “selective” and 26% of the 43 “inclusive” schools do. Public institutions are significantly more likely to offer population health educational programs than private institutions, with 51% of the 96 public schools and 30% of the private schools offering a major and/or minor. This difference could be attributable to differences in enrollment size. A full 90% (162/180) of the small schools and 96% (46/48) of the very small schools in the sample are private. Urban institutions are significantly more likely than other schools to offer population health programs. While 45% of institutions located in cities offer a major and/or minor, only 31% in towns, 25% in suburbs, and 10% in rural areas do. This trend is also related to enrollment size. While 100% of the 21 institutions in rural areas are small or very small, only 38% of the 188 institutions in cities are small or very small.

### Global health and public health

**Figure 2:**
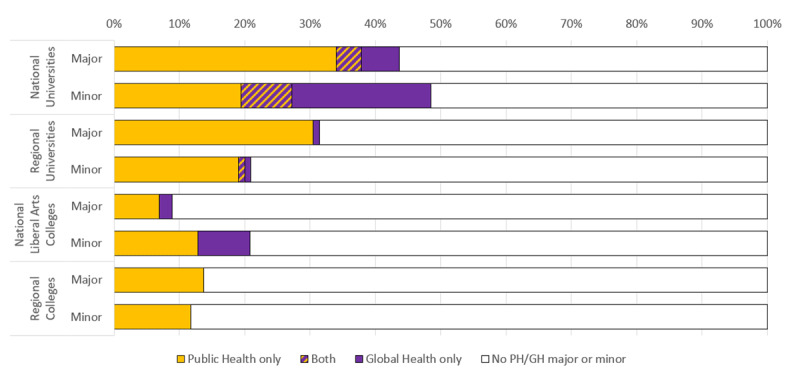
Distribution of schools offering majors and minors in public health (PH) (orange) and global heath (GH) (purple) offerings, by institutional type during the 2019–20 academic year.

Public health programs are more common than global health programs, but some schools offer studies in both areas (Figure 2). Of the 101 institutions with population health majors, 92 have a public health major, 13 have a global health major, and 4 have both. Of the 105 institutions with population health minors, 74 offer a public health minor, 40 a global health minor, and 9 both. Most of the institutions with global health programs are doctoral degree granting universities. Fifty of the 103 national universities in our sample offer population health minors, including 28 offering a public health minor, 30 offering a global health minor, and 8 offering both. Master’s universities offer more public health programs than liberal arts colleges, but liberal arts colleges offer more global health programs than master’s universities. Of the 101 liberal arts colleges in our sample, 21 offer a population health minor, including 13 offering public health minors and 8 offering global health minors.

## Discussion

Our analysis shows that majors and minors in public health, global health, and other population health areas are offered by diverse institutions in the United States. While these educational programs are most common at large universities, schools of all types, sizes, and locations are offering majors and minors in areas related to population health. Population health studies can support the educational mission of diverse types of institutions of higher education by promoting development of competencies in communication, information literacy, critical thinking, ethical decision-making, research methods, teamwork, and leadership and integrating coursework across the natural sciences, the social and behavioral sciences, math and quantitative reasoning, and the humanities and fine arts [1,14,15,16]. Population health curricula enable students to understand health from a diversity of perspectives and provide opportunities for internships, study abroad, service learning, integrative capstone projects, and other types of experiential learning that stimulate personal growth and enhance résumés and graduate school applications [17].

The sizeable proportion of schools in our sample that offer educational programs in population health areas is further evidence that these fields have become popular areas of study among undergraduate students in the United States [4]. The rising demand for these majors and minors is part of a larger pattern of increased interest in health studies at the undergraduate level. Nationally, health professions and related programs—a category that includes public health studies and four-year clinical degrees, such as nursing—is now the second most popular undergraduate degree area after business. The percentage of bachelor’s degrees awarded for majors in the health professions rose from 6.1% of the total in 2000 to 11.9% in 2015 [9]. Considerably more degrees are now awarded each year in the health professions (#2) than in social sciences and history (#3), psychology (#4), biological and biomedical sciences (#5), and engineering (#6) [9]. Health studies alumni with only baccalaureate degrees have higher-than-average salaries compared to their bachelor’s-only peers [10]. A higher-than-average percentage of individuals who earn undergraduate health degrees go on to earn graduate and medical degrees, and health-related majors who go on to earn graduate degrees earn more than their graduate-degree holding peers in other fields [10].

Public health is the most popular major and minor within the population health area. One of the reasons for the popularity of public health education is that it provides a foundation for a variety of career pathways. Undergraduate public health programs that are accredited by CEPH or have adopted their recommended curricular domains and foundational competencies prepare students directly for employment in public health roles. Other public health programs are designed to enable students to meet the Certified Health Education Specialist (CHES) eligibility requirements, prepare for other types of professional certifications, and/or complete the courses required for admission to medical, dental, and other postgraduate clinical schools. Courses and concentrations in public health subdisciplines such as epidemiology, environmental health, and health communication also equip students with skills and competencies that can be directly applied in work settings.

Global health programs are especially common at large universities and at liberal arts colleges. At universities with graduate public health programs, undergraduate global health education is sometimes presented as a subdiscipline of public health. At other schools, global health curricula are often designed as multidisciplinary programs outside of traditional public health boundaries that emphasize interdisciplinary, integrative approaches to understanding population health. Smaller liberal arts colleges may not employ full-time professors who have completed specialized training in a population health area, but these schools often have faculty members whose research and teaching interests overlap with global health issues. Moving forward, it may be helpful for a national or international group to develop competencies for undergraduate and graduate global health education that define the scope of the field as an area of academic study and identify the distinct goals for global health education at the bachelor’s and master’s levels [11,12,13].

The main limitation of our study is that the colleges and universities that are highly ranked by *U.S. News & World Report* tend to be ones that have competitive admissions processes, so they are not representative of all schools within their institutional categories. Only a little over one-quarter of all bachelor’s-degree granting institutions are included in our analysis. However, it is reasonable to expect that the general trends—such as population health education programs being more common at larger universities than at smaller colleges—would remain even if the sample was expanded. The strength of our analysis is that it includes national and regional universities and colleges rather than focusing on just one of these groups. A secondary limitation is that our analysis focuses on public health and global health majors and minors rather than using a more generous definition for what counts as a population health program. This conservative approach may have undercounted the number of population health programs currently being offered. To overcome this potential shortcoming in our analysis, the results section also presents some analysis of majors and minors that are closely related to public and global health, such as environmental health and health communication.

This analysis shows that public health and global health have become common areas of study at the bachelor’s level in the United States. Population health programs were initially offered primarily at large universities and selective liberal arts colleges [5,6,7], and they remain popular at these types of institutions, but majors and minors in population health areas are now offered by schools with diverse profiles. Programs at different types of institutions will encounter unique opportunities and challenges related to curricular offerings and design, facultyrecruitment and development, student retention, experiential learning opportunities, and graduate placement. Further exploration of the learning objectives, competencies, and curricula for population health programs will provide insights about current practices and emerging approaches for excellence in undergraduate education. Clarity about the goals of undergraduate and graduate educational pathways in public health, global health, and other population health areas will enable colleges and universities to make informed decisions about which types of educational programs are the best match to their institutional missions, resources, strengths, and limitations.
